# Global analysis on the mutations associated with multidrug-resistant urogenital mycoplasmas and ureaplasmas infection: a systematic review and meta-analysis

**DOI:** 10.1186/s12941-023-00627-6

**Published:** 2023-08-10

**Authors:** Mohammad Abavisani, Masoud Keikha

**Affiliations:** 1 Student research committee, Mashhad University of Medical Sciences, Mashhad, Iran; 2 Department of Medical Microbiology, School of Medicine, Iranshahr University of Medical Sciences, Iranshahr, Iran

**Keywords:** Drug resistance, Mutation, Mycoplasma infections, Ureaplasma infections

## Abstract

**Background:**

The emergence of multidrug-resistant (MDR) strains of genital pathogens, notably *Mycoplasma genitalium* and *Ureaplasma* spp., constitutes a significant global threat today. The present study aimed to evaluate the prevalence and trend of changes in MDR *mycoplasma* and *ureaplasma* strains.

**Methods:**

An exhaustive search was performed across the ISI Web of Science, PubMed, Scopus, ScienceDirect, and Google Scholar databases to accumulate relevant studies without restrictions until April 2023. We used event rate and corresponding 95% confidence intervals to determine the frequency of resistance-related mutations and examine the trend of antibiotic resistance changes.

**Results:**

The data from 27 studies, including 24,662 patients across 14 countries, were evaluated. Out of the total studies, 20 focused on *M. genitalium* infections, and five on *Ureaplasma* spp. The frequency of resistance-associated mutations to macrolides, tetracyclines, and fluoroquinolones in clinical strains of *M. genitalium* was 43.5%, 13.1%, and 18.6%, respectively. The prevalence of *M. genitalium* strains with double resistance and MDR was 11.0% and 17.4%, respectively. The incidence of both double-drug-resistant and MDR strains was higher in the World Health Organization (WHO) Western Pacific Region than in European and American populations. For *Ureaplasma* strains, resistance-associated mutations to macrolides, tetracyclines, and fluoroquinolones were 40.8%, 25.7%, and 90.3%, respectively. The rate of antibiotic resistance was higher in the African population compared to the European and WHO Western Pacific Regions. The rate of MDR *Ureaplasma* infections was 13.2%, with a higher incidence in the African population compared to the WHO Western Pacific and European regions.

**Conclusion:**

The proliferation and spread of MDR *Mycoplasma* and *Ureaplasma* strains present a significant public health challenge. The situation is indeed alarming, and the rising trend of MDR *M. genitalium* and MDR *Ureaplasma* infections suggests that therapies involving macrolides and fluoroquinolones may become less effective.

## Background

In response to the escalating issue of antibiotic resistance among infectious agents, a high-priority health crisis concern, the World Health Organization (WHO) recently initiated a project entitled “Global health sector strategies on HIV, viral hepatitis, and sexually transmitted infections (STIs) for the period 2022–2023” [1]. Especially troublesome are microorganisms like mycoplasmas and ureaplasmas, which are STIs associated with the *Mollicutes* class and the *Mycoplasmataceae* family. These pathogens quickly develop resistance to antimicrobial agents, propagating antibiotic resistance within their populations [2]. *Mycoplasma genitalium* was first isolated from the urethral swabs of two males exhibiting non-gonococcal urethritis (NGU) symptoms in 1980 [3]. Due to the bacterium’s fastidious nature and cultivation difficulties, its pathogenic role remained ambiguous for several years. Now, *M. genitalium* is recognized as a crucial sexually transmitted pathogen, infecting or colonizing over 3% of the global population [4]. This particular pathogen is associated with NGU in men and conditions such as endometritis, salpingitis, cervicitis, pelvic inflammatory disease, and preterm birth in women [5, 6].

Given *M. genitalium*’s lack of peptidoglycan in its cell wall, it exhibits resistance to β-lactam antibiotics. Antibiotics that disrupt protein synthesis and DNA synthesis inhibitors are the most effective treatment options currently available for *M. genitalium* infections [4]. Until early 2016, when the status of macrolide resistance was still undefined, the International Union against STI (IUSTI) European guidelines recommended doxycycline 100 mg twice daily or 200 mg once daily orally for seven days [7]. However, early randomized controlled clinical trials indicated that azithromycin was superior to doxycycline [8]. Consequently, most regional and international guidelines now consider azithromycin (as a one-time dose of 1 g) as the primary treatment for *M. genitalium* infections, and moxifloxacin (400 mg daily for seven days) as the secondary treatment [9]. Nonetheless, numerous studies reveal a rising resistance to macrolides and fluoroquinolones in *M. genitalium* isolates, with macrolide resistance exceeding 50% in some regions [10]. Fluoroquinolone resistance can reach 20% in the Asia-Pacific region, increasing treatment failure rates to more than 30% of cases [11–15]. The emergence of multidrug-resistant (MDR) *M. genitalium* strains and cases of co-infection with *Chlamydia trachomatis* further complicate treatment [4, 16].

Ureaplasmas, free-living pathogens like *Ureaplasma parvum* (UPA) and *Ureaplasma urealyticum* (UUA), invade the lower urogenital tract in approximately 20–29% of healthy women [17]. Infections with these agents increase the risk of adverse pregnancy outcomes such as miscarriage, stillbirth, chorioamnionitis, and preterm labor [18, 19]. Clinical samples from patients with urethritis, endometritis, chronic prostatitis, and bacterial vaginosis often isolate genital ureaplasmas. These infections elevate the risk of infertility in couples [20]. Moreover, these pathogens can cause severe illnesses in infants, including pneumonia, bacteremia, meningitis, and chronic lung disease [21]. Ureaplasmas, like mycoplasmas, lack a cell-wall structure, making β-lactam antibiotics and glycopeptides ineffective against these pathogens; unfortunately, these organisms are intrinsically resistant to lincosamides such as clindamycin [22]. Consequently, the treatment of *Ureaplasma* infections is restricted to bacteriostatic agents like protein synthesis inhibitors (e.g., tetracyclines and macrolides) and DNA replication inhibitors (e.g., fluoroquinolones). However, resistance to these antibiotics is rising due to the creation of single nucleotide polymorphisms (SNPs) and the spread of resistance genes [23, 24]. Although antibiotic resistance in ureaplasmas has been observed globally, the resistance burden varies geographically due to differences in antibiotic consumption and histories of previous antimicrobial exposure. However, data on the antibiotic resistance pattern of ureaplasmas remain sparse in many parts of the world, especially in economically disadvantaged nations. Developing appropriate treatment strategies necessitates understanding and evaluating current antibiotic resistance trends [25].

The decline in the efficacy of antibiotic regimens against mycoplasmas and ureaplasmas signifies an emerging public health crisis. Surveillance and analysis of resistance rates are critical steps towards optimizing guidelines for treating these infections effectively. In this study, we assess the rate and trend of dual resistance and MDR in *mycoplasma* and *ureaplasma* infections and measure primary treatment failure in cases of infection with these strains.

## Methods

Throughout the course of this research, all the steps encompassing literature search in databases, selection criteria establishment, data extraction, and statistical analysis were rigorously conducted in line with the guidelines proposed by the Preferred Reporting Items for Systematic Reviews and Meta-Analyses (PRISMA) standard [26].

### Literature search

A systematic search was executed across various databases such as ISI Web of Science, PubMed, Scopus, Science Direct, and Google Scholar to gather publications appraising the resistance of mycoplasmas and ureaplasmas to macrolides, tetracycline, and fluoroquinolones. Until April 2023, our search methodology utilized MeSH keywords such as “*Mycoplasma*”, “*M. genitalium*”, “*M. pneumonia*”, “*M. hominis*”, “*M. salivarium*”, “*Ureaplasma*”, “*U. parvum*”, “*U. urealyticum*”, “Drug resistant”, “Multi-drug resistant”, “MDR”, “Macrolides”, “Tetracyclines,“ and “Fluoroquinolones”. This phase was completed by two separate authors, disregarding language or publication date limitations. In order to ensure comprehensive coverage, we manually verified the bibliographies of the included studies to gather all pertinent articles.

### Selection criteria

To examine studies that assessed multiple antibiotic resistances of mycoplasmas and ureaplasmas in clinical samples, two independent authors participated; disagreements were resolved through deliberation between these authors. All gathered papers were imported into Endnote software. The titles, abstracts, and full texts of all articles were scrutinized, and those meeting our pre-defined criteria were deemed eligible. Our inclusion criteria included: (1) Articles that evaluated antibiotic resistance mutations in clinical isolates of mycoplasmas and ureaplasmas, including 2058 and 2059 mutations in the 23 S rRNA, S83L, S83R, D87N, and D87Y mutations in *parC*, and all mutations in *gyrA*, *gyrB*, and *parE* genes, (2) Publications discussing antibiotic resistance in mycoplasmas and ureaplasmas, (3) Original studies adopting cross-sectional, case-control, and longitudinal designs, (4) Research articles based on human clinical samples containing clinical DNA samples and/or culture-based DNA samples, (5) Studies utilizing standard consensus microbiological methodologies. Conversely, in vitro or animal model publications, duplicate studies, articles with incomplete results, and non-original articles were excluded.

### Quality assessment and data extraction

The Joanna Briggs Institute’s (JBI) critical appraisal checklist was employed to evaluate the quality of the studies. This checklist assesses indicators such as population size, research objectives, sample collection method, and statistical analysis approach, assigning scores to each element. Any study achieving at least six points was included in the analysis. As indicated in Table 1, data concerning the first author, study location, study year, gender distribution of patients, type of infections, patient symptoms, antibiotic resistance detection method, infection rate, resistance rate to macrolide, tetracycline, and fluoroquinolones, MDR rate, and primary treatment failure rate were extracted from eligible studies.

**Table 1:** Characteristics of included studies

First author	Country	Year	Patients		Type of infection	Type of infection	Diagnostic method	Infection rate (%)	Resistance to (%)				Primary treatment failure	Ref
			Men	Women					macrolide	tetracycline	fluoroquinolone	DMF/MDR		
Gamova	Russia	2003	NR	NR	CUID	*Ureaplasma* spp.	MIC	NR	23.1	19.5	NR	1.6	NR	41
Tagg	Australia	2013	NR	NR	Urethritis, cervicitis, PID	*M. genitalium*	Sequencing	76.88	33.56	NR	9.09	9.79	NR	51
Kikuchi	Japan	2014	NR	NR	NR	*M. genitalium*	Sequencing	56.6	25	NR	29.41	17.64	NR	50
Deguchi	Japan	2015	0	149	Sex workers	*M. genitalium*	Sequencing	14.1	47.1	NR	36.8	25.0	NR	45
Deguchi	Japan	2016	NR	NR	NR	*M. genitalium*	Sequencing	NR	7.01	NR	23.46	26.31	NR	37
Zhu	China	2016	7374	0	Reproductive disorders	*M. hominis*	MIC	0.4	NR	NR	NR	33.78	NR	39
Zhu	China	2016	7374	0	Reproductive disorders	*Ureaplasma* spp.	MIC	42.3	NR	NR	NR	1.09	NR	39
Roy	France	2016	NR	NR	NR	*M. genitalium*	Sequencing	NR	17.20	NR	6	1.2	NR	46
Murray	Australia	2017	112	43	NR	*M. genitalium*	Sequencing	90.32	NR	NR	13.6	8.6	34.83	47
Barbera	Spain	2017	62	20	Urethritis, PID	*M. genitalium*	Sequencing	NR	35	NR	8	4.1	13.51	28
Xiao	USA	2017	158	0	Urethritis (HIV/MSM)	*M. genitalium*	Real-time PCR	17.1	70.37	NR	29.62	18.51	NR	29
Shipitsyna	Russia	2017	NR	NR	Urethritis, cervicitis	*M. genitalium*	Sequencing	NR	5.30	NR	6	1.04	NR	33
Odom	UK	2018	157	0	Urethritis (HIV/MSM)	*M. genitalium*	Real-time PCR	17.2	74.07	NR	29.62	24	NR	31
Hamasuna	Japan	2018	148	0	Urethritis	*M. genitalium*	Real-time PCR	NR	20.52	NR	35.09	12.5	2.64	32
Mulligan	Ireland	2019	400	0	Urethritis, asymptomatic (HIV/MSM)	*M. genitalium*	Real-time PCR	3	75	NR	33.3	33.3	NR	34
Xiao	USA	2019	58	58	Urethritis, cervicitis	*M. genitalium*	Real-time PCR	12.1	60.7	NR	11.1	11.1	NR	35
Boujemaa	Tunisia	2020	NR	NR	Infertile, gynecological infections	*Ureaplasma* spp.	MIC	9.55	100	37.62	100	37.62	NR	36
Biyik	Turkey	2020	NR	NR	asymptomatic	*Ureaplasma* spp.	MIC	43.1	39.3	46.4	96.4	35.7	NR	43
Dumke	Germany	2021	56	2	Urethritis, asymptomatic	*M. genitalium*	Real-time PCR	NR	56.9	6.8	10.3	6.9	6.7	30
Ma	China	2021	33	225	Urethritis, cervicitis, PID	*Ureaplasma* spp.	Sequencing	NR	32.95	17.44	89.92	15.12	NR	40
Nijhuis	Netherlands	2021	811	1781	NR	*M. genitalium*	Real-time PCR	4.1	40.6	NR	8.1	7.6	NR	49
Braam	Netherlands	2022	550	119	Urethritis, cervicitis, asymptomatic	*M. genitalium*	Real-time PCR	NR	62	NR	8.6	6.87	2	38
Xiao	USA	2022	NR	NR	Invasive infections	*M. salivarium*	MIC	NR	100	14.3	25.7	14.3	NR	42
Day	UK	2022	NR	NR	STI clinic	*M. genitalium*	RT-PCR	17.75	71	NR	8	7	NR	44
Baetselier	Belgium	2022	148	64	Urethritis, Cervicitis, asymptomatic	*M. genitalium*	RT-PCR	NR	74.3	NR	39.7	31.3	NR	48
Li	China	2023	60	149	Urethritis, cervicitis, PID, asymptomatic	*M. genitalium*	SAT	1.30	64	22.5	67.5	56.3	28.29	27
Edelstein	Russia	2023	170	43	NR	*M. genitalium*	Sequencing	NR	25.82	NR	17.37	7.04	NR	52

**Abbreviations**: CUID, chronic urogenital inflammatory diseases; DMF, Dual macrolide and fluoroquinolone resistance; MIC, micro inhibitory concentration; NR, not reported; PID, pelvic inflammatory disease; SAT, real-time probe simultaneous amplification and testing.

### Statistical analysis

We utilized the Comprehensive Meta-Analysis (CMA) software version 2.2 (Biostat, Englewood, NJ, USA) to compile data and evaluate the global burden of MDR infections in mycoplasmas and ureaplasmas. Furthermore, the incidence of dual resistance to macrolides and fluoroquinolones in human infections was investigated. In cases of significant heterogeneity (I2 index greater than 50, Cochrane p-value greater than 0.05), a random-effects meta-analysis was employed to generate overall estimates. The data was further divided into subgroups according to WHO geographical regions, time periods (2003–2015, 2016–2019, 2020–2023), and men who have sex with men (MSM). Sensitivity analyses were performed using the leave-one-out approach to minimize heterogeneity. Primary treatment failure in MDR infections, along with instances of dual macrolide and fluoroquinolone resistance, were also evaluated. The funnel plot’s asymmetry was examined to assess potential publication bias.“

## Results

### Literature search and study characteristics

**Fig. 1:**
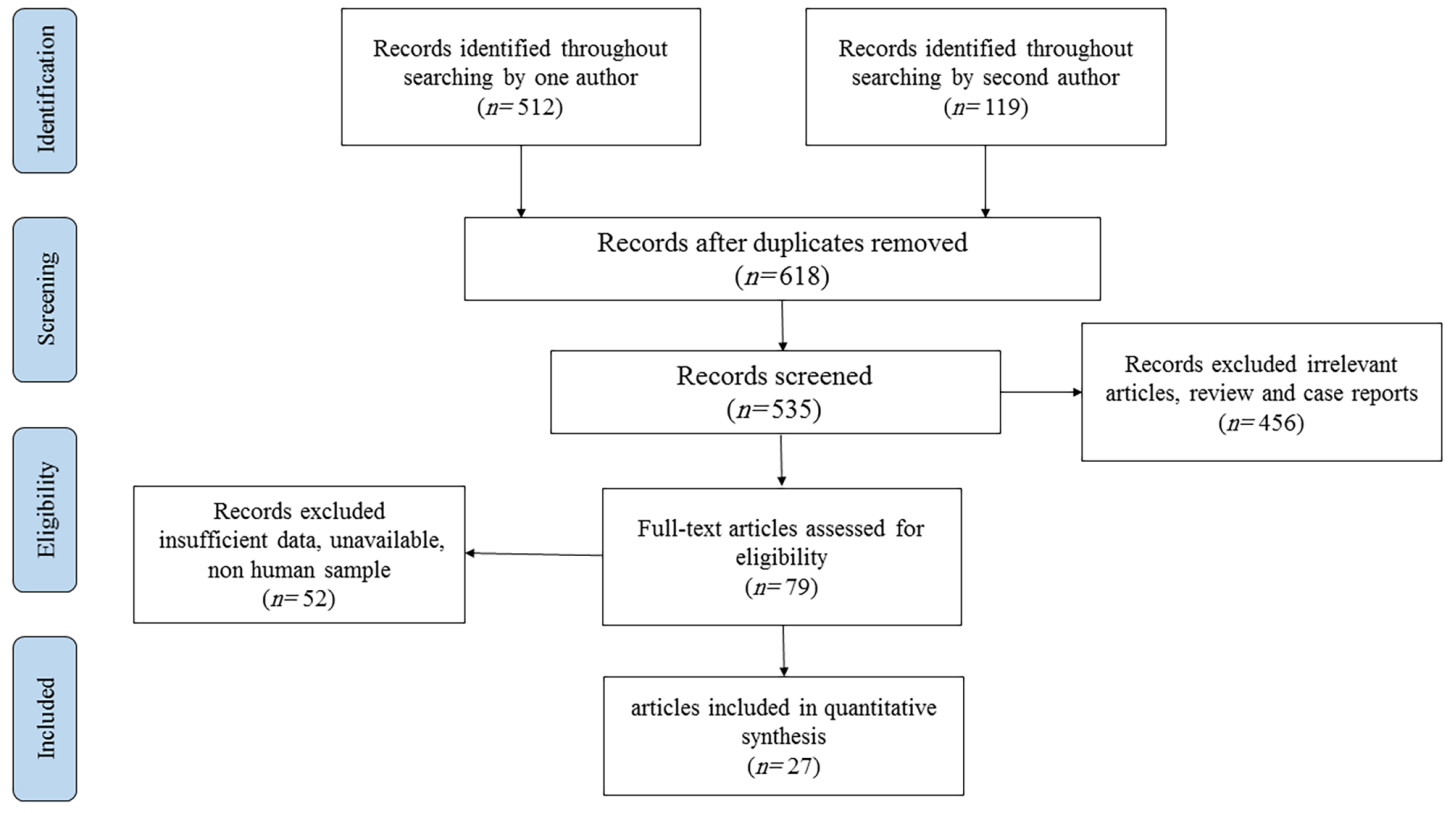
The flowchart of included studies

As illustrated in Fig. 1, a database search yielded 631 articles. Upon review of titles and abstracts, 456 studies were deemed irrelevant to our criteria and thus excluded. The full text of the remaining 79 articles was examined, and coupled with studies included from reference list reviews, a total of 27 studies were determined to be eligible for inclusion [20, 27–51].

Table 1 presents the salient features of these selected studies. A total of 27 studies involving 24,662 patients from 14 disparate countries were analyzed. Patients exhibiting symptoms such as NGU, cervicitis, pelvic inflammatory disease, chronic urogenital inflammatory diseases, invasive infections, and infertility, along with asymptomatic partners, were evaluated for *mycoplasma* and *ureaplasma* infections. Of the total participants, 86.94% were male, while the remaining 13.06% were female. Furthermore, four additional studies assessed *M. genitalium* infection and antibiotic resistance among HIV-positive MSM. Among the included studies, nine were conducted in European countries, three in the WHO Western Pacific region, and one each in the WHO Americas and African regions. Twenty investigations focused on *M. genitalium* infections, five on *Ureaplasma* spp., one on *M. hominis*, and one on *M. salivarium*.

To diagnose *mycoplasma* and *ureaplasma* infections, the studies employed various methods, including culture, serology, and molecular techniques. Antibiotic resistance in mycoplasmas and ureaplasmas was ascertained using broth microdilution, Sanger sequencing, Reverse transcription polymerase chain reaction (RT-PCR), real-time PCR, and real-time probe simultaneous amplification and testing. Potential sources of bias for the included studies could include lack of random selection, low and diverse population sizes in some studies, variety in clinical samples, and differences in methods used to diagnose *mycoplasma* and *ureaplasma* infections. Some studies also evaluated infections among stored banks of samples. Despite these potential biases, the infection rate for *M. genitalium* and *Ureaplasma* spp. in the included studies was estimated to be 21.0% (95% CI: 10-38.9) and 28.9% (95% CI: 13.0-52.4), respectively. Moreover, the rate of *M. genitalium* infection in MSM individuals was estimated to be around 11.5% (95% CI: 5.3–23.1).

### Characteristics of mycoplasmas antibiotic resistance

**Fig. 2:**
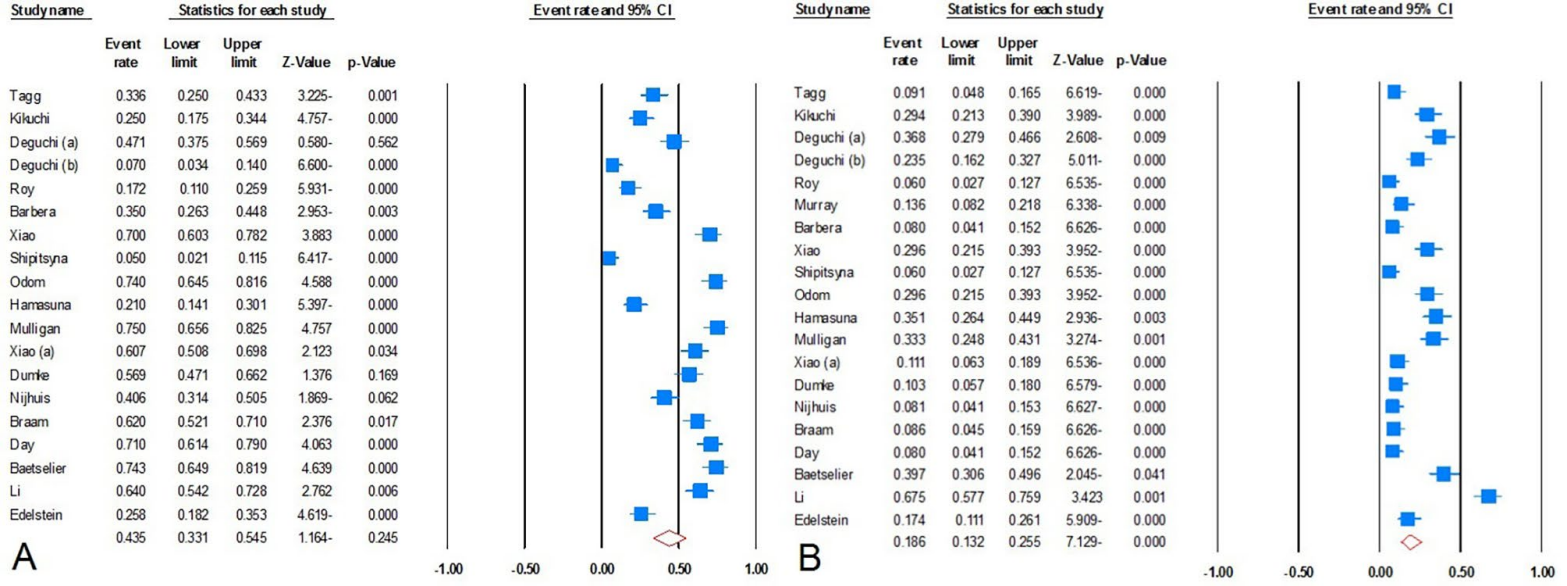
Meta-analysis on the prevalence of mutations associated with *M. genitalium* resistance to antibiotics; **(A)** macrolides, **(B)** fluoroquinolones

We conducted a comprehensive analysis of data from 20 trials encompassing 9058 patients spanning 12 countries between the years 2013 and 2023. The prevalence of mutations linked to *M. genitalium* resistance to macrolides, tetracyclines, and fluoroquinolones was found to be 43.5% (95% CI: 33.1–54.5), 13.1% (95% CI: 3.8–36.8), and 18.6% (95% CI: 13.2–25.5), respectively, as demonstrated in Fig. 2.

The incidence of mutations linked to *M. genitalium* resistance to macrolides in European countries was 47.1% (95% CI: 32.7–61.9), 30.3% (95% CI: 17.2–47.6) in the WHO Western Pacific region, and 65.2% (95% CI: 58.3–71.5) in the WHO Americas region. Moreover, mutations associated with macrolide resistance were discerned in 70.9% of MSM individuals (95% CI: 66.2–75.1). The overall estimates corroborate both the regional and temporal trends. Our findings indicate that the prevalence of mutations tied to macrolide resistance has surged in recent years, manifesting in 25.6% (95% CI: 13.3–43.6) during 2003–2015, 41.5% (95% CI: 62.2–23.4) during 2016–2019, and 56.6% (95% CI: 43.5–68.9) during 2020–2023. During the subgroup analysis focused on mutations related to tetracycline resistance, the prevalence of mutations linked to *M. genitalium* resistance to tetracyclines in European regions and the WHO Western Pacific region was recorded as 6.8% (95% CI: 3.2–13.7) and 22.5% (95% CI: 15.4–31.7), respectively. Based on the statistical analysis, the prevalence of mutations associated with resistance was 13.1% (95% CI: 3.8–36.8) between 2020 and 2023. Unfortunately, due to data insufficiency, we were unable to discern trends in the mutations associated with tetracycline resistance in previous periods.

In terms of mutations connected with fluoroquinolone resistance, the prevalence in European countries, the WHO Western Pacific region, and the United States was 13.6% (95% CI: 8.3–21.3), 28.4% (95% CI: 16.9–43.7), and 19.0% (95% CI: 6.7–43.5), respectively. Globally, the trend of mutations associated with fluoroquinolone resistance decreased from 23.2% (95% CI: 11.2–42.0) in 2003–2015 to 17.6% (95% CI: 11.9–25.4) in 2016–2019, with 18.3% (95% CI: 7.6–37.7) remaining relatively stable until 2020–2023. *M. salivarium* antibiotic resistance mutations for macrolides, tetracyclines, and fluoroquinolones were reported to be 100%, 14.3%, and 25.7%, respectively. Recent observations of dual resistance to macrolides and fluoroquinolones in clinical isolates of *M. genitalium* have amplified concerns regarding treatment failure with both options. The incidence of mutations linked to resistance to two drugs in the WHO Western Pacific region was found to be about 14.6% (95% CI: 8.7–23.6), which exceeded that in European regions (10.0%; 95% CI: 4.7–20.0) and America (4.1; 95% CI: 0.4–32.3). According to our trend analysis, the frequency of resistance has tapered off in recent years, with a non-significant decline from 17.1% (95% CI: 10.1–27.4) in 2003–2015 to 7.2% (95% CI: 3.2–15.1) in 2016–2019, and 11.1% (95% CI: 4.1–26.7) in 2020–2023.

Regrettably, the prevalence of mutations linked to MDR *M. genitalium* strain infection escalated from 18.5% (95% CI: 10.9–29.6) in 2016–2019 to 32.6% (95% CI: 26.0–40.0) in 2020–2023, a trend that is disconcerting. The global rate of MDR *M. genitalium* infection was estimated to be 17.4% (95% CI: 9.2–30.5); the rate of MDR strain infection in the WHO Western Pacific region exceeded that in the European region (29.1% (95% CI: 10.7–58.4) vs. 12.2% (95% CI: 5.3–25.5), respectively). Sensitivity analyses, performed subsequent to the identification of the source of variability, revealed no significant changes in pooled values. For *M. salivarium* and *M. hominis*, the rates of infection with MDR strains were determined to be 14.3% and 33.78%, respectively. Unfortunately, only a single study evaluated the multiple resistances of these two pathogens, thereby precluding the calculation of the infection rate and trend.

Our findings highlighted that the prevalence of MDR *M. genitalium* in the MSM population was approximately 19.2% (95% CI: 8.7–37.2). Furthermore, the rate of primary treatment failure in MDR *M. genitalium* infections was measured at 12.8% (95% CI: 6.9–22.6). Thus, the rate of primary treatment failure in MDR *M. genitalium* isolates among WHO western Pacific patients was about 18.1% (95% CI: 7.1–38.9), consistent with the rate observed in the European population (13.2% (95% CI: 6.3–25.7)). Gratifyingly, the global trend of MDR *M. genitalium* infection between 2016 and 2019 was in the same range as 2020–2023 (19.7% (95% CI: 11.2–32.2) vs. 18.1 (95% CI: 13.3–24.2)).

### Characteristics of ureaplasmas antibiotic resistance

**Fig. 3:**
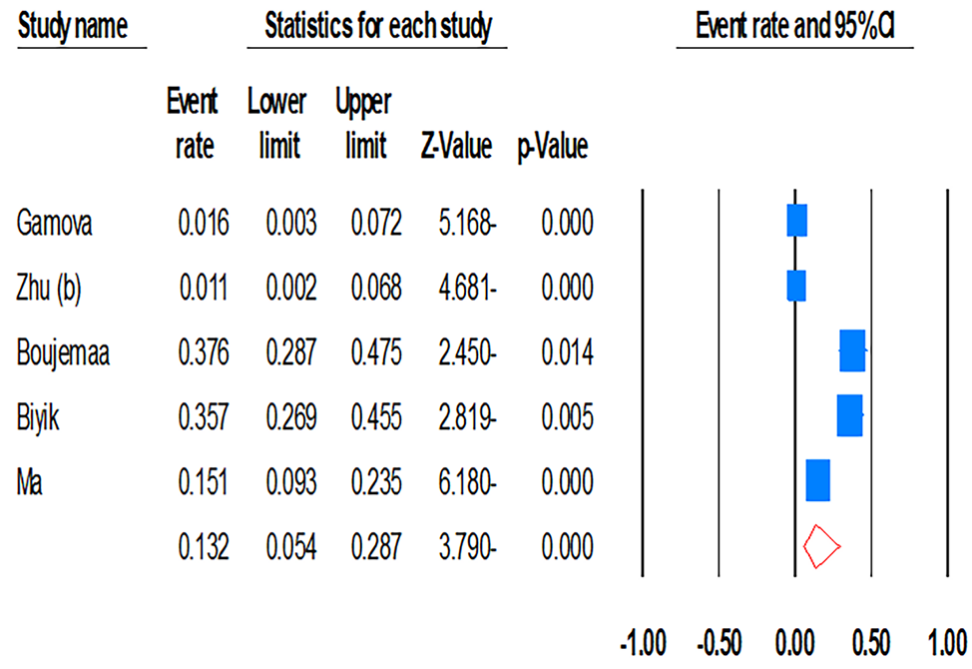
Meta-analysis on the prevalence of clinical isolates of MDR-*Ureaplasma* spp. Infections

This comprehensive quantitative analysis incorporates data from five articles, which include 7826 clinical samples sourced from four different countries, spanning the years 2003 to 2021. The pooled prevalence of mutations associated with resistance to three antibiotics—macrolides, tetracyclines, and fluoroquinolones—was calculated as 40.8% (95% CI: 23.6–60.7), 25.7% (95% CI: 15.4–39.6), and 90.3% (95% CI: 43.1–99.1), respectively (Fig. 3).

In the African region, the rate of mutations connected with macrolide resistance in clinical isolates of ureaplasmas was near total at 99.5% (95% CI: 92.6–100). Comparatively, in European countries and the WHO Western Pacific region, this rate was considerably lower at 30.8% (95% CI: 17.3–48.6) and 33.0% (95% CI: 24.5–42.7), respectively. Between 2020 and 2023, mutations associated with macrolide resistance were prevalent in 52.9% of cases (95% CI: 27.3–77.1). A notable rise in mutations tied to tetracycline resistance was observed in recent years, surging from 19.5% (95% CI: 12.9–28.4) in 2003 to 32.8% (95% CI: 18.5–51.1) in 2023. The rate of mutations resistant to this antibiotic was estimated at 31.6% (95% CI: 11.7–61.7) in Europe, 17.4% (95% CI: 11.2–26.1) in the WHO Western Pacific, and 37.6% (95% CI: 28.7–47.5) in African regions. The prevalence of fluoroquinolone resistance is particularly concerning. In Europe, this was measured at 96.4% (95% CI: 90.3–98.7), in the Western Pacific it was 89.9% (95% CI: 82.3–94.5), and in Africa, it reached 99.5% (95% CI: 92.6–100). In the period from 2020 to 2023, the resistance rate of ureaplasmas was 95.6% (95% CI: 85.8–98.8), suggesting that fluoroquinolones have become ineffective for treating human *Ureaplasma* infections worldwide. The data insufficiently cover temporal trends in other regions or time periods. Notwithstanding the substantial heterogeneity, sensitivity analyses indicated that summary values were stable.

The global rate of MDR *Ureaplasma* infection was assessed to be 13.2% (95% CI: 5.4–28.7). The prevalence of MDR *Ureaplasma* strains was approximately 9.4% (95% CI: 0.3–76.6) in WHO European regions, 4.9% (95% CI: 0.3–43.7) in the WHO Western Pacific regions, and 37.6% (95% CI: 28.7–47.5) in African regions. Alarmingly, the trend of MDR *Ureaplasma* infection has escalated sharply from 1.6% (95% CI: 0.3–7.2) in 2003–2015 to 28.6% (95% CI: 16.8–44.3), signaling a serious concern for health authorities. Regrettably, due to insufficient information, we could not calculate the rate of primary treatment failure among MDR *Ureaplasma* infections.

### Publication bias

**Fig. 4:**
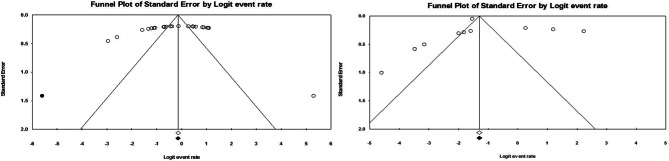
Funnel plots of meta-analysis of eligible studies centered at corresponding 95% confidence intervals

Our study’s funnel plots exhibited asymmetry in certain instances, suggesting a notable bias in some overall estimates. Nevertheless, the instances of significant asymmetry were assessed using the trim-and-fill method. This method, applied across all cases, revealed no substantial or meaningful alterations to the summary estimates (as depicted in Fig. 4).

## Discussion

Presently, the ongoing emergence and steady proliferation of antibiotic resistance in bacterial agents implicated in both simple and complex forms of urogenital infections, notably mycoplasmas and ureaplasmas, has triggered a persistent decline in the efficacy of treatments for these infections, thereby posing a global challenge. This has been underscored by two extensive epidemiological studies that have sounded the alarm about the escalating prevalence of antibiotic resistance in *M. genitalium*, especially within European territories [52, 53].

Based on these studies, macrolide resistance in *M. genitalium*, with a particular focus on azithromycin resistance, is attributed to point mutations occurring in the V region of the 23s rRNA gene at positions 2058 and 2059, as per *Escherichia coli* numbering [54, 55]. Additionally, amino acid modifications in *parC*, namely S83L, S83R, D87N, or D87Y, are frequently correlated with failed ciprofloxacin therapy [32, 56]. Within the scope of this systematic review and meta-analysis, we examined the prevalence of antibiotic resistance mutations in clinical isolates of *M. genitalium* and ureaplasmas, making a novel contribution to the field by quantifying the MDR burden and tracking its geographical variations for the first time. As per our analysis, the collective prevalence of mutations associated with resistance to macrolides, tetracyclines, and fluoroquinolones was estimated at 43.5%, 13.1%, and 18.6% respectively. Our analysis of resistance to tetracyclines in *M. genitalium* is based on only two reports, which may not reflect the true prevalence and diversity of resistance mechanisms in such bacterium. Therefore, we caution that more studies are needed to confirm and expand our findings. Our scrutiny of the temporal trajectory of macrolide resistance revealed a marked surge in resistance, increasing from 25.6% in the period 2003–2015 to 56.6% in 2020–2023. Given that single-dose azithromycin has been commonly adopted as the primary treatment for STIs in various global regions since the early 1990s, it is anticipated that the escalating trend of mutations related to macrolide resistance in *M. genitalium* will precipitate a continual decline in the annual success rate of treatments [57–59]. Supporting this conjecture, a meta-analysis by Lau et al. found a steady rise over time in the treatment failure rates of *M. genitalium* infections administered with azithromycin, mirroring the trend identified in our analysis [60]. This annual increase in macrolide resistance can largely be attributed to the absence of drug sensitivity tests for *M. genitalium*, the implementation of inappropriate treatment protocols, and the dearth of large-scale surveillance to gauge the antibiotic resistance of *M. genitalium*.

Our study indicates that the prevalence of mutations linked to macrolide resistance is more pronounced in the American and European regions compared to the WHO Western Pacific region. The discrepancy in resistance levels across various geographical areas can primarily be traced back to the differences in antimicrobial usage, treatment guidelines, and the diversity of local antimicrobial stewardship programs implemented in these areas. For instance, doxycycline is typically designated as the frontline treatment in countries with low macrolide resistance, while azithromycin tends to be the first choice in nations that demonstrate substantial resistance to this class of antibiotics [33, 61]. Further analysis of our data revealed that the incidence of mutations related to macrolide resistance in clinical strains of *M. genitalium* isolated from MSM significantly surpasses that found in heterosexual men, at a rate of 70.9%. This upswing in macrolide resistance within this demographic can be ascribed to asymptomatic infections of this pathogen and the recurrent use of azithromycin to treat frequent instances of *chlamydia* and *Neisseria gonorrhoeae* infections [62]. This notion is substantiated by numerous studies conducted within the MSM community, which have identified a high prevalence of macrolide resistance in syphilis and gonorrhea infections [63, 64]. Interestingly, our results showed a higher rate of tetracycline resistance in the WHO Western Pacific countries than in European regions. Current research suggests that tetracycline is extensively utilized in Asian regions, resulting in a considerable resistance burden to tetracyclines in Southeast Asian territories [65, 66]. With significant Asian populations migrating to Australia and the WHO Western Pacific regions, the resistance burden in these areas is further exacerbated. Consequently, the emergence of tetracycline resistance should be anticipated in clinical isolates from the WHO Western Pacific region.

Our analysis demonstrates that the occurrence of mutations linked to fluoroquinolone resistance in *M. genitalium* samples from the WHO Western Pacific population is approximately twice to thrice the resistance burden found in European and American regions. This area also exhibits the highest rate of gonorrhea resistance to fluoroquinolones [67, 68]. Most of the studies incorporated in our analysis originated from Japan, where fluoroquinolones such as levofloxacin, gatifloxacin, and moxifloxacin are commonly employed in treating NGU and *M. genitalium* infections [32]. Also other studies demonstrated that sitafloxacin could be an alternative to moxifloxacin as an antimicrobial agent for a second-line treatment of *M. genitalium* infections unsuccessfully treated with azithromycin regimens [69, 70]. As a consequence, the frequency of mutations connected to fluoroquinolones is anticipated to be higher in the WHO Western Pacific region than in the European region.

Our study also discovered that the rate of fluoroquinolone-related mutations has remained relatively stable over the past 16 years, indicating no significant shifts in resistance. These findings align with those reported by Machalek and colleagues [15]. Additionally, our study shows a higher rate of dual resistance and MDR *M. genitalium* in the WHO Western Pacific populations compared to the WHO European populations. This rise in resistance mirrors the rate of antibiotic misuse, history of antimicrobial exposure, and treatment guidelines for STIs in the WHO Western Pacific region, particularly in Japan and China. It also contributes to an increase in MDR in clinical isolates of *M. genitalium* [4, 16]. It is intriguing to note that the rates of MDR *M. genitalium* are higher in the MSM community than in other patient demographics. We infer that MSM patients bear a higher MDR burden than heterosexual men, primarily due to the frequent exposure to azithromycin because of co-infection of *M. genitalium* with *chlamydia* or *N. gonorrhea*. Regrettably, the trend of MDR *M. genitalium* infection has been on the rise in recent years, and if the current scenario persists, the efficacy of these antibiotic classes in treating *M. genitalium* infection will be lost. Consequently, utilizing innovative technologies for simultaneous pathogen detection and resistance biomarker identification could lead to more precise resistance status evaluations and the selection of the most effective initial treatment for these infections [71, 72]. Also with considering only a single study, we concluded that the rate of infection with MDR *M. hominis* was determined to be 33.78%. in another study, midrange resistance rates for *M. hominis* to tetracycline, doxycycline and minocycline were 50.0%, 9.0% and 16.7%, respectively [73]. Because of the relationship of *M. hominin* with genital infections and the importance of the issue, more investigations must be conducted to determine antibiotic resistance status more precisely [74]. In our study, we have discerned that the prevalence of infection involving MDR *M. salivarium* stands at 14.3%. Despite the long-held assumption regarding the non-pathogenic nature of *M. salivarium*, it has been found that this bacterium is a common inhabitant of dental plaque and gingival sulci [75, 76]. Moreover, while extra-oropharyngeal infections involving *M. salivarium* are considered rare, *M. salivarium* has also been identified in infections at other body sites [77–79]. Despite these findings, the understanding of the antibiotic resistance characteristics of *M. salivarium* remains limited, with only a single study having evaluated its multidrug resistance profile to date. Therefore, there is a pressing need for additional research that focuses on exploring the antibiotic resistance of this bacterium.

Likewise, *ureaplasma* resistance has become widespread in many global regions due to the overuse of antibiotics and the implementation of empirical therapies without drug susceptibility testing [80]. *Ureaplasma* spp. are divided into 14 different serovars across two species, UUA and UPA. Current studies show that the trend towards increasing minimum inhibitory concentration (MIC) is greater in UUA than in UPA [81, 82]. According to our research, the rate of mutations connected to resistance to macrolides and fluoroquinolones was 52.8% (95% CI: 22.8–81.0) and 97.2% (95% CI: 92.8–98.9), respectively. However, the prevalence of resistance-related mutations in *Ureaplasma* spp. was 40.8% for macrolides, 25.7% for tetracyclines, and 90.3% for fluoroquinolones. Hence, our findings align with prior research demonstrating that the antibiotic resistance status in UUA is more severe than in other pathogens in this family (UPA) [83, 84]. Also, Ahmadi MH, operating a meta-analysis study, calculated the midrange resistance rates for UUA/UPA to tetracycline, doxycycline, and minocycline as 43.3%, 28.6%, and 9.0%, respectively. The related maximum resistance rates were 86.5, 57.1, and 16.2, respectively [73]. Our research revealed a high prevalence of macrolide resistance burden in clinical isolates of ureaplasmas isolated from African patients. Due to widespread antibiotic misuse in African populations and the absence of a comprehensive surveillance system to monitor antibiotic resistance status in this region, the antibiotic resistance status in these regions is typically more severe than in other parts of the world [85, 86].

According to our findings, the rate of mutations associated with tetracycline resistance in *Ureaplasma* spp. isolates from African regions was higher than in the WHO European and Western Pacific regions. Tetracyclines are commonly used as the first line of treatment for *Ureaplasma* infections in African regions, and thus it is logical to presume that the tetracycline resistance burden in these regions is higher than the global average [87]. Unfortunately, the number of mutations linked to tetracycline resistance has been steadily increasing, primarily due to empirical therapy without assessing the antibiotic resistance pattern of *Ureaplasma* spp. isolates.

Our analysis also revealed a high level of fluoroquinolone resistance across the globe, with the pooled prevalence of mutations linked to fluoroquinolone resistance in Europe, the WHO Western Pacific, and Africa being 96.4%, 89.9%, and 99.5%, respectively. The widespread use of fluoroquinolones to treat various infectious diseases can lead to high fluoroquinolone resistance [23]. MDR *Ureaplasma* infection was found in 13.2% of the population, and the prevalence of MDR *Ureaplasma* spp. strains in the African population is much higher than in the European and WHO Western Pacific populations. Prior research supports our findings, indicating that *Ureaplasma* antibiotic resistance is higher in developing countries than in developed ones [88]. Regrettably, the global trend of MDR *Ureaplasma* has risen from 1.6% to 2013 to 28.6% in 2023. The excessive use of antibiotics and a lack of local antimicrobial resistance surveillance contribute to an uncontrollable increase in antibiotic resistance.

As a result, the present study assists clinicians in refining treatment guidelines to circumvent and manage the emergence of antibiotic-resistant strains by illuminating the epidemiological traits of MDR *M. genitalium* and *Ureaplasma* strains. Despite this, our study has certain limitations; (1) Our investigation assessed the prevalence of antibiotic resistance mutations in samples from symptomatic patients, hence, our findings have implications for STI clinics, but cannot be generalized to the wider population, (2) Despite our utilization of a random-effects model, high heterogeneity was observed. Various factors such as clinical, methodological, and statistical can contribute to this heterogeneity in pooled estimates. Clinical heterogeneity can arise due to characteristics such as participant characteristics, socioeconomic status, body mass index, gender, age, symptoms, HIV status, infection site, and mutation identification methods. Statistical heterogeneity may lead to inconsistent results in subgroup analyses; sensitivity analysis might not significantly reduce the heterogeneity, (3) The WHO American and African regions have been underrepresented in the research, with no studies conducted in Southeast Asian or Eastern Mediterranean nations, (4) Our research only identified a few mutations for resistance to macrolides and fluoroquinolones, hence, we did not investigate mutations outside these regions associated with resistance and treatment failure, (5) Despite the inclusion of research focusing on various *Mycoplasma* species in our investigative analysis, only two studies pertaining to *M. hominis* and *M. salivarium* met the eligibility criteria. Consequently, to apply these findings more broadly to other *Mycoplasma* species, additional research must be conducted to validate and support these interpretations. Lastly, despite our re-evaluation and adjustment of the pooled estimates of bias instances using the trim-and-fill method, significant publication bias was observed in our selected research.

## Conclusion

The increasing prevalence of antibiotic resistance in *Mycoplasma* and *Ureaplasma* infections is a significant yet underappreciated challenge; if unchecked, it may escalate into a global problem. Current treatment guidelines for *Mycoplasma* and *Ureaplasma* largely rely on anecdotal evidence rather than drug susceptibility testing. Our results underscore the need to reevaluate the use of empirical antibiotic therapy with single-dose azithromycin for the treatment of these infections. Minimizing the use of macrolides and fluoroquinolones, employing resistance-guided treatment guidelines, adopting combination therapy with new classes of antimicrobial agents, implementing simultaneous detection methods for infection and antibiotic resistance mutations, and carrying out epidemiological surveillance of antibiotic resistance can aid clinicians not only in effectively treating *Mycoplasma* and *Ureaplasma* treatment-resistant infections but also in mitigating the rise in antibiotic resistance.

## Data Availability

All data generated or analyzed during this study are included in this published article.

## Abbreviations

*M. genitalium*: *Mycoplasma genitalium*
MDR: Multidrug-resistant
MSM: Men who have sex with men
WHO: World Health Organization
NGU: Non-gonococcal urethritis
UPA: *Ureaplasma parvum*
UUA: *Ureaplasma urealyticum*
SNPs: Single nucleotide polymorphisms
PRISMA: Reporting Items for Systematic Reviews and Meta-Analyses
JBI: Joanna Briggs Institute’s
CMA: Comprehensive Meta-Analysis
CI: Confidence interval
